# Neuroimaging of hypophysitis: etiologies and imaging mimics

**DOI:** 10.1007/s11604-023-01417-y

**Published:** 2023-04-03

**Authors:** Ryo Kurokawa, Mariko Kurokawa, Akira Baba, Moto Nakaya, Shimpei Kato, Jayapalli Bapuraj, Yasuhiro Nakata, Yoshiaki Ota, Ashok Srinivasan, Osamu Abe, Toshio Moritani

**Affiliations:** 1 Division of Neuroradiology, Department of Radiology, University of Michigan, 1500 E Medical Center Dr, Ann Arbor, MI 48109 USA; 2 Department of Radiology, Graduate School of Medicine, The University of Tokyo, 7-3-1, Hongo, Bunkyo-ku, Tokyo, 113-8655 Japan; 3 Department Or Neuroradiology, Tokyo Metropolitan Neurological Hospital, 2-6-1 Musashidai, Fuchu, Tokyo, 183-0042 Japan

**Keywords:** Hypophysitis, Magnetic resonance imaging, PitNET

## Abstract

Hypophysitis is an inflammatory disease affecting the pituitary gland. Hypophysitis can be classified into multiple types depending on the mechanisms (primary or secondary), histology (lymphocytic, granulomatous, xanthomatous, plasmacytic/IgG4 related, necrotizing, or mixed), and anatomy (adenohypophysitis, infundibulo-neurohypophysitis, or panhypophysitis). An appropriate diagnosis is vital for managing these potentially life-threatening conditions. However, physiological morphological alterations, remnants, and neoplastic and non-neoplastic lesions may masquerade as hypophysitis, both clinically and radiologically. Neuroimaging, as well as imaging findings of other sites of the body, plays a pivotal role in diagnosis. In this article, we will review the types of hypophysitis and summarize clinical and imaging features of both hypophysitis and its mimickers.

## Introduction

Hypophysitis is a potentially life-threatening inflammatory disease of the pituitary gland accounting for 0.24–0.88% of all intra- and suprasellar lesions [1]. Hypophysitis can be classified into categories depending on the mechanisms (primary or secondary), histology (lymphocytic, granulomatous, xanthomatous, plasmacytic/IgG4 related, necrotizing, or mixed), and anatomy (adenohypophysitis, infundibulo-neurohypophysitis, or panhypophysitis). Secondary causes of hypophysitis include systemic granulomatous diseases, infection, sellar tumors or cysts, and drugs [2]. Clinically, patients with hypophysitis present with inflammatory symptoms such as headaches and vomiting, and varying degrees of hypopituitarism. Although the diagnosis of hypophysitis ultimately requires a biopsy, the presumptive diagnosis based on clinical manifestation in conjunction with magnetic resonance imaging (MRI) and laboratory findings is typically made in clinical settings [2]. Early assessment of treatment response of hypophysitis on pituitary MRI with serial imaging until clear stability has been recommended [1]. Furthermore, the exclusion of other potential mimickers is necessary for appropriate management. In this article, we review the clinical and imaging features of primary and secondary hypophysitis, as well as various types of mimickers.

## Etiology and imaging features of hypophysitis

### Lymphocytic hypophysitis

**Fig. 1:**
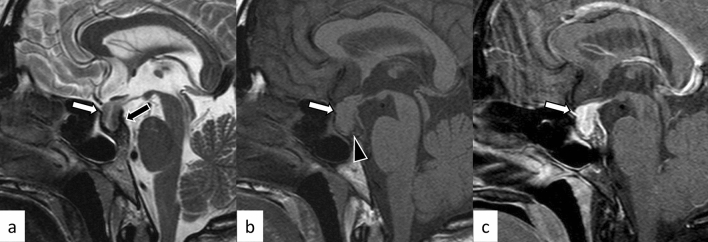
A 38-year-old woman with lymphocytic hypophysitis. An enlarged pituitary gland shows heterogeneous hyperintensity on T2-weighted sagittal image (**a**, white arrow), isointensity on T1-weighted sagittal image (**b**, white arrow), and homogeneous contrast enhancement (**c**, white arrow). A dark signal intensity area is observed on the sellar floor (“parasellar T2 dark sign”; **a**, black arrow). The posterior pituitary T1-weighted bright spot is absent (**b**, arrowhead). The different images of this case were evaluated in the previous study [9]

**Fig. 2:**
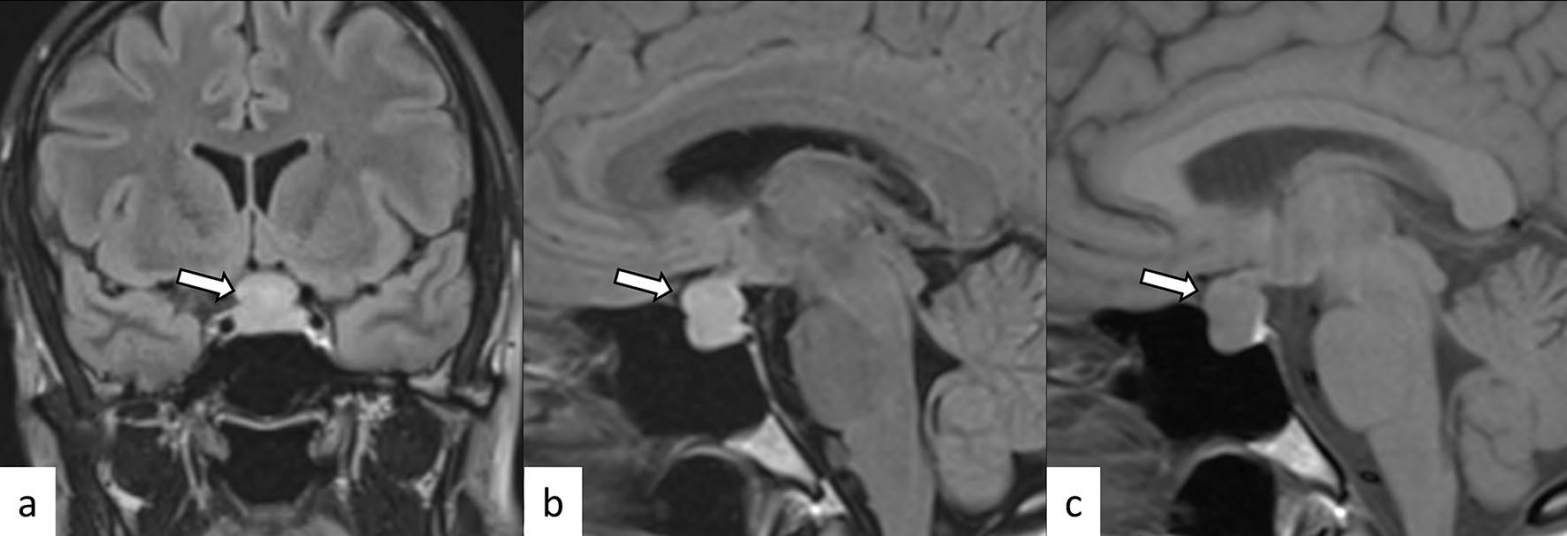
A 37-year-old woman with lymphocytic hypophysitis. A “snowman-like” shaped enlarged pituitary gland is observed (**a**–**c**, arrows). The pituitary gland shows homogeneous hyperintensity on fluid-attenuated inversion recovery (FLAIR) coronal and sagittal images (**a**, **b**) and homogeneous isointensity on T1-weighted sagittal image (**c**). The posterior pituitary T1-weighted bright spot is preserved in this case

**Fig. 3:**
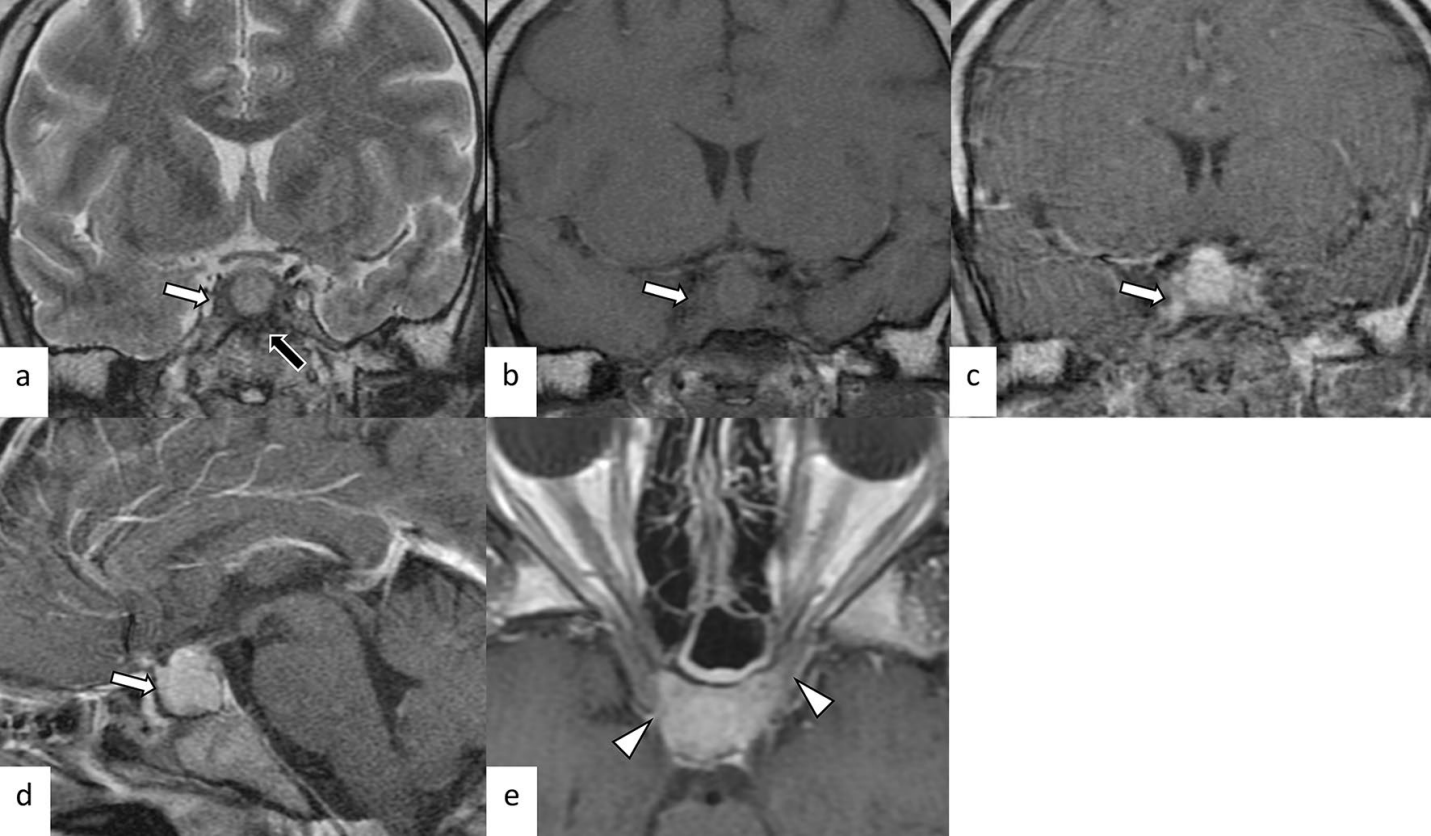
A 29-year-old man with primary granulomatous hypophysitis. Artifacts due to the patient’s body motion are observed. An enlarged pituitary gland shows heterogeneous hyperintensity on T2-weighted coronal image (**a**, white arrow), isointensity on T1-weighted coronal image (**b**, white arrow), and homogeneous contrast enhancement on post-contrast T1-weighted coronal and sagittal images (**c**, **d**, white arrows). Extension into bilateral cavernous sinus is observed (**e**, arrowheads). T2-weighted hypointensity areas around the enlarged pituitary gland are found, suggestive of granulomatous/fibrous inflammation (**a**, black arrow)

Lymphocytic hypophysitis is the most common type of primary hypophysitis, accounting for 76–86% of cases [3]. Lymphocytic hypophysitis is histopathologically characterized by lymph follicles of T and B lymphocytes forming lymph follicles and fibrosis [3, 4]. It is thought to be due to autoimmune mechanisms and is more frequent during pregnancy and in the early postpartum period [5]. Development in association with other autoimmune diseases such as Graves’ disease, Hashimoto’s disease, and Addison’s disease has been reported [5]. Clinically, central diabetes insipidus/arginine vasopressin deficiency (48–72%), hypogonadism (60–80%), adrenal dysfunction (71%), and hypothyroidism (81%) are frequently observed [6–8]. High-dose corticosteroids are the mainstay of treatment, but spontaneous regression is also frequent [4]. Surgical procedure is occasionally indicated in refractory cases, in those with enlarged gland exhibiting mass effect on adjacent critical structures, and in those with visual field defects. Other than pituitary gland enlargement, Nakata et al. [9] reported loss of posterior pituitary T1-weighted bright spot (17/20, 85%), enlarged pituitary stalk (17/20, 85%), and homogeneous contrast enhancement (13/19, 68%) were typical MR imaging findings of lymphocytic hypophysitis. The parasellar T2 dark sign was noted in 35% of cases, which the authors reported was helpful in differentiating lymphocytic hypophysitis and pituitary neuroendocrine tumor (PitNET) (Figs. 1, 2). It should be noted, however, that the parasellar T2 dark sign can also be observed in other chronic inflammatory diseases besides lymphocytic hypophysitis (Fig. 3). Sato et al. reported that abnormal enhancement of the pituitary gland in lymphocytic hypophysitis was delayed to over 90 s, even though a conventional MRI showed a normal pituitary, highlighting the usefulness of dynamic contrast-enhanced MRI [10].

### Granulomatous hypophysitis

**Fig. 4:**
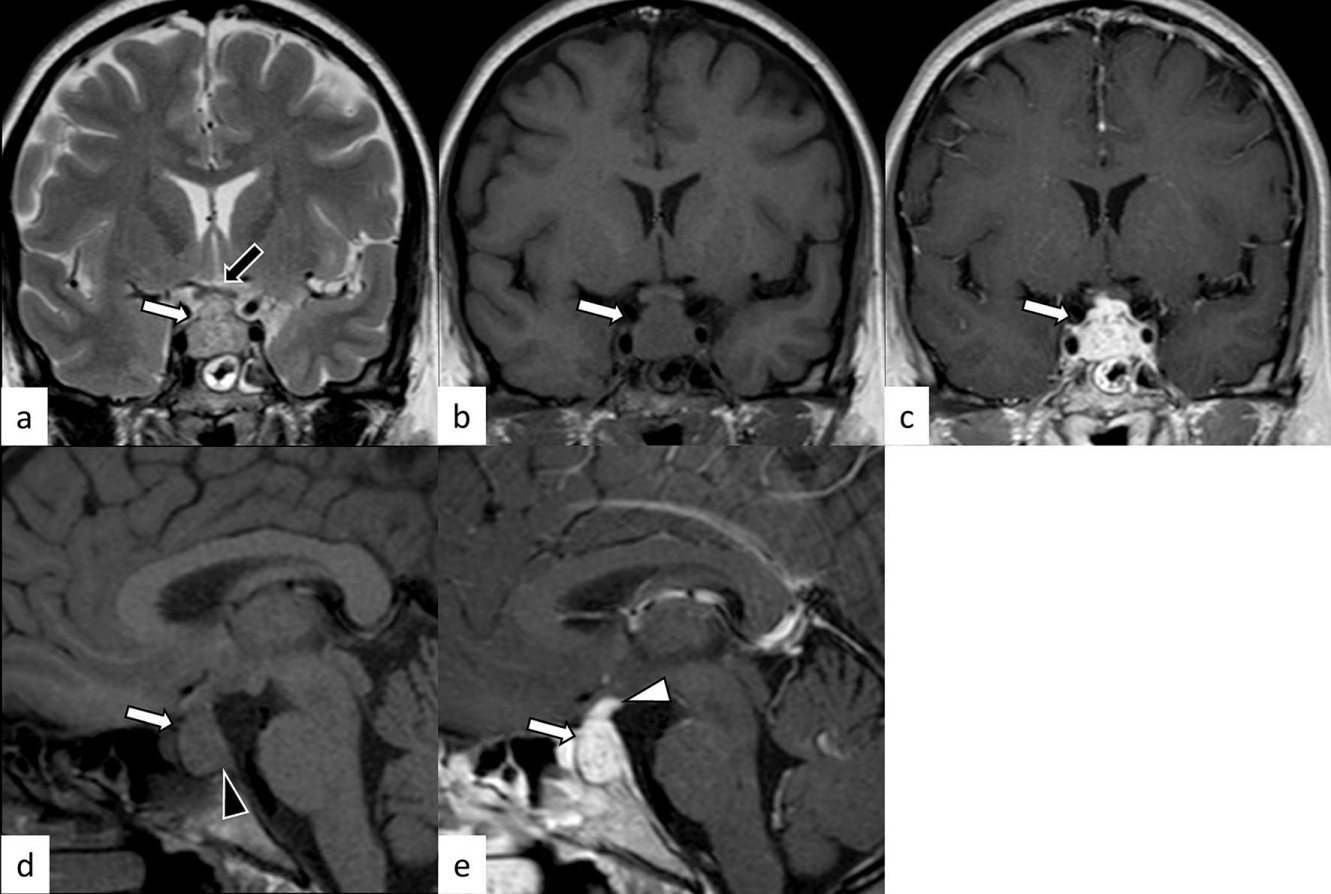
A 45-year-old woman with secondary granulomatous hypophysitis associated with Crohn’s disease. Both pituitary gland and stalk are enlarged (**a**–**e**, white arrows; e, white arrowhead), abutting the optic chiasm superiorly (**a**, black arrow). The pituitary gland shows heterogeneous hyperintensity on T2-weighted coronal image (**a**), hypointensity on T1-weighted coronal image (**b**), and heterogeneous vivid enhancement (**c**, **e**). The posterior pituitary T1-weighted bright spot is absent (**d**, black arrowhead)

A review article showed that granulomatous hypophysitis accounts for 20% of all primary hypophysitis [3]. It can also occur secondary to systemic granulomatous diseases. Granulomatous hypophysitis is histopathologically characterized by large numbers of multinucleated giant cells and histiocytes aggregated as granulomas. The primary form mainly affects women, and the average age at onset is 43 years [3]. Endocrine dysfunctions involve the growth hormone (21/30, 70%), adrenocorticotropic hormone (ACTH)/cortisol (38/52, 73%), gonadotropins (35/53, 66%), thyroid-stimulating hormone (TSH)/T4 (35/54, 65%), and prolactin (35/54, 65%) [11]. Diabetes insipidus/arginine vasopressin deficiency (22/82, 27%) is also an important correlate [11]. Cranial nerve symptoms involving the optic nerve (12/82, 15%), oculomotor nerve (12/82, 15%), abducens nerve (6/82, 7%), and facial nerve (2/82, 2%) were also observed [11]. Granulomatous hypophysitis is considered less responsive to corticosteroid therapy compared with lymphocytic hypophysitis, and surgery and long-term hormone replacement are frequently required [3, 6, 11]. Secondary causes of granulomatous hypophysitis include multiple types of diseases, including infection (tuberculosis, syphilis, and fungus), autoimmune diseases (sarcoidosis, granulomatosis with polyangiitis (GPA), Takayasu arteritis, Cogan’s syndrome, and Crohn’s disease), histiocytosis (Langerhans cell histiocytosis (LCH) and Erdheim–Chester disease), and specific sellar and suprasellar lesions (Rathke’s cleft cyst, PitNET, germinoma, and craniopharyngioma) [3]. MR imaging findings are not significantly different from those of lymphocytic hypophysitis (Figs. 3, 4) [6]; however, loss of T1-weighted bright spot in the posterior pituitary lobe is less frequent (10/51, 20%), and the contrast enhancement tends to be more heterogeneous [6, 11]. The preoperative edema and contrast enhancement of the infrasellar basisphenoid bone marrow have been reported as differentiating MRI findings of granulomatous hypophysitis from PitNET [12], although further accumulation of cases are needed to determine whether these MRI findings are also found in other types of hypophysitis.

### Plasmacytic/IgG4-related hypophysitis

**Fig. 5:**
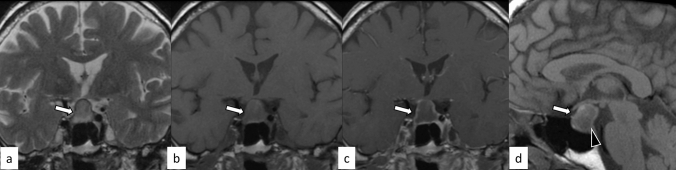
A 67-year-old man with plasmacytic/IgG4-related hypophysitis. An enlarged pituitary gland shows heterogeneous hyperintensity on T2-weighted coronal image (**a**, arrow) and hypointensity on T1-weighted coronal image (**b**, arrow) with peripheral enhancement and loss of central enhancement (**c**, arrow). The posterior pituitary T1-weighted bright spot is absent (**d**, black arrowhead)

**Fig. 6:**
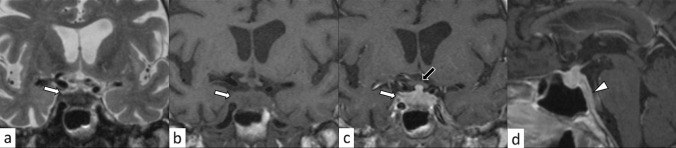
A 76-year-old woman with plasmacytic/IgG4-related hypophysitis. An enlarged strongly enhanced pituitary gland and stalk are observed (**a**–**c**, white arrows). The lesion shows heterogeneous hypointensity on T2-weighted coronal image **a** and hypo- to isointensity on T1-weighted coronal image **b** with involvement of the right cavernous sinus and compression of the optic chiasm (**c**, black arrow). Inflammatory extension along the dorsal surface of the clivus is observed (**d**, arrowhead)

Plasmacytic/IgG4-related hypophysitis accounts for up to 30% of all primary hypophysitis [3, 13]. Histopathologically, plasmacytic/IgG4-related hypophysitis is characterized by lymphocyte and plasma cell-rich mononuclear infiltration of the pituitary gland, with storiform fibrosis and > 10 IgG4-positive cells/high-power field [3]. Pituitary involvement is found in 1.7–8% of patients with IgG4-related disease, although plasmacytic/IgG4-related hypophysitis can occur without the involvement of other sites [14]. When the pituitary gland is the only involved site, the diagnosis of IgG4-related disease cannot be made, according to the 2019 American College of Rheumatology/European League Against Rheumatism classification criteria [15, 16]. Frequently encountered symptoms include anterior hypopituitarism (86/102, 84%), diabetes insipidus/arginine vasopressin deficiency (71/89, 80%), and bitemporal hemianopia (19/31, 61%) [14]. While the response to corticosteroid therapy is good, recovery of endocrine dysfunction is considered rare [6]. Besides the nonspecific MRI findings of hypophysitis, such as pituitary enlargement, abnormal enhancement, and loss of T1-weighted bright spot in the posterior pituitary lobe (Figs. 5, 6), associated heterogeneous T2-weighted signal intensity and enhancement may be observed. The involvement of IgG4-related diseases in the salivary glands, lacrimal glands, meninges, and cranial nerves can be diagnostic indicators if present. There have been reports of empty sella after plasmacytic/IgG4-related hypophysitis [17]. The pattern of uptake and multi-organ involvement on 18F-fluorodeoxyglucose positron emission tomography (18F-FDG PET) can be diagnostic clues of plasmacytic/IgG4-related hypophysitis when other typical metabolic lesions are demonstrated [1].

### Xanthomatous hypophysitis

**Fig. 7:**
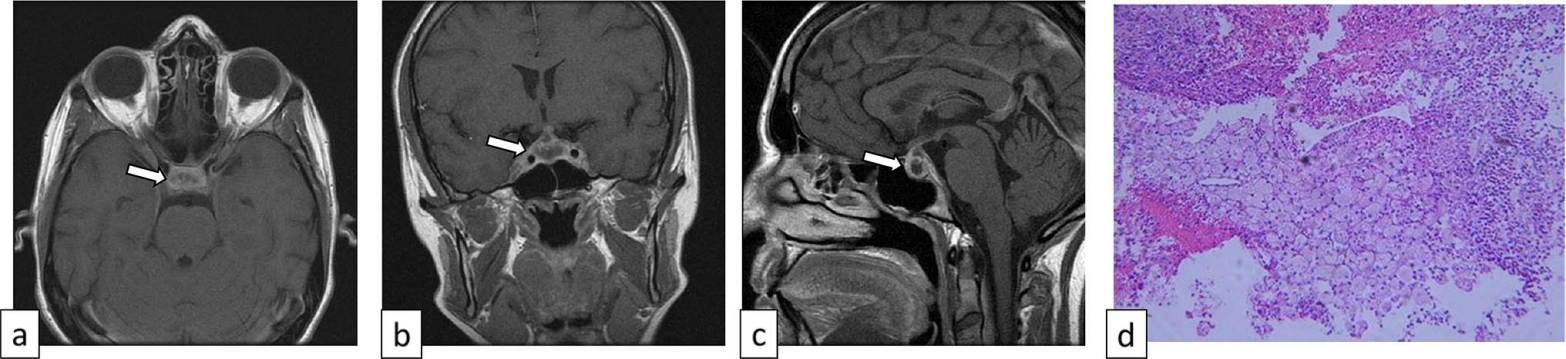
A 31-year-old woman with xanthomatous hypophysitis. Post-contrast T1-weighted images show an enlarged pituitary gland with central non-enhancing components and peripheral contrast enhancement (**a**–**c**, arrows). Pathology shows fragments of intact, normal pituitary gland infiltrated by foamy histiocytes (**d**). The images were cited and modified from [19]

Xanthomatous hypophysitis is the second rarest type of hypophysitis, accounting for 3% of all primary hypophysitis [3]. Histopathologically, xanthomatous hypophysitis is characterized by the infiltration of foamy histiocytes without granuloma formation, with fibrosis in the chronic phase [3]. Researchers suggested that at least some cases of xanthomatous hypophysitis develop giant cell reaction (xanthogranulomatous hypophysitis) in association with Rathke’s cleft cyst leakage/rupture/hemorrhage [18]. The average age at onset is 34 years, with 19:7 female-to-male predominance [18]. Clinically, symptoms resemble those of mild lymphocytic hypophysitis, and optic tract-related symptoms have been infrequently reported [6]. Xanthomatous hypophysitis is thought to be less responsive to corticosteroid therapy, and endocrine dysfunctions tend to be difficult to ameliorate. On MRI, xanthomatous hypophysitis tends to have cystic components, and peripheral contrast enhancement is observed (Fig. 7) [19]. The cystic components on MRI correspond to a dark orange fluid-filled cyst with suspended crystals seen macroscopically [6].

### Necrotizing hypophysitis

**Fig. 8:**
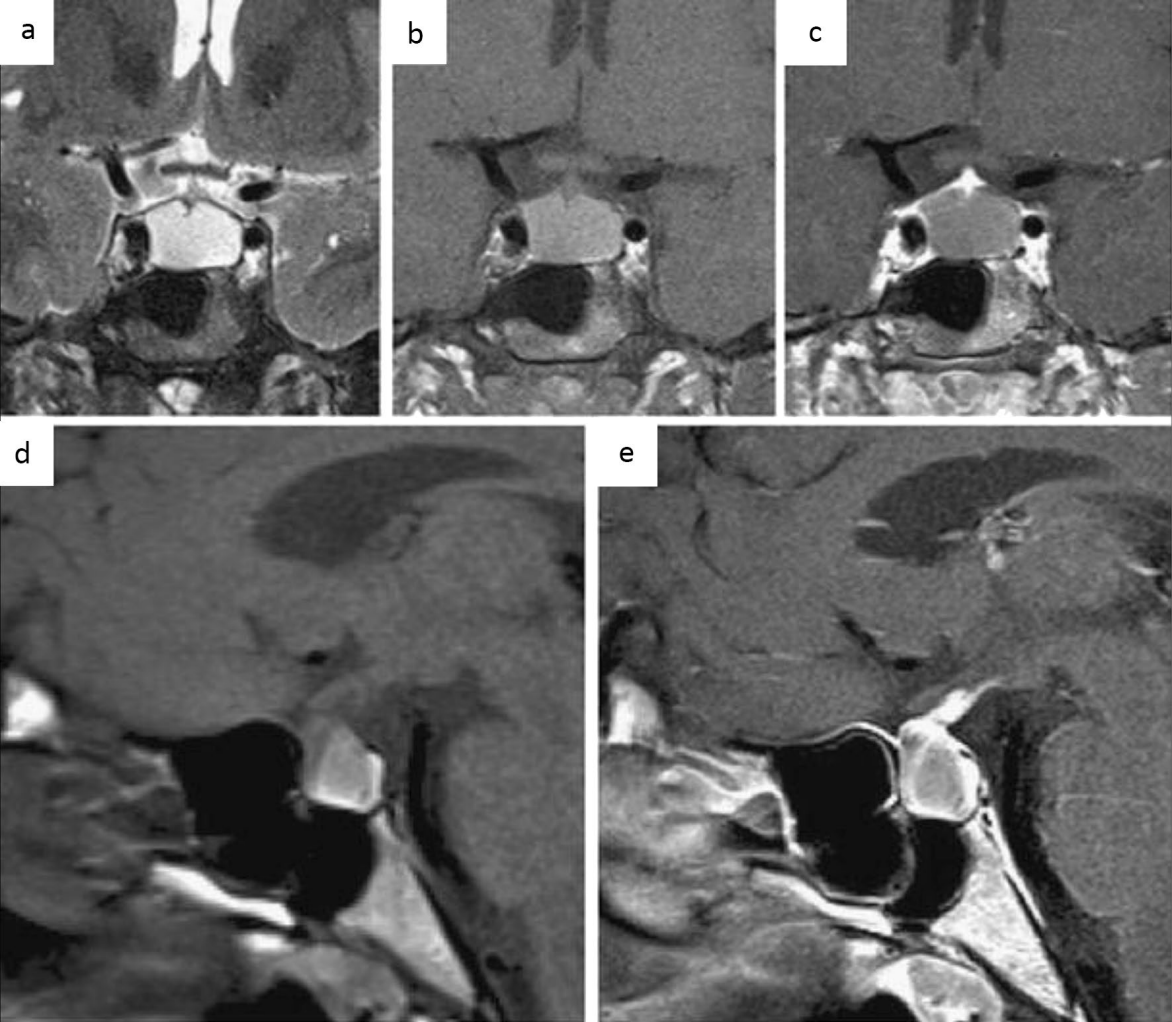
A 20-year-old woman with necrotizing hypophysitis. The enlarged pituitary gland and stalk showing homogeneous hyperintensity on T2- and T1-weighted coronal images **a**, **b** with an absence of central contrast enhancement and strong peripheral enhancement **c**, **e** are observed. The posterior pituitary T1-weighted bright spot is absent (**d**). The images were cited from [21]

Necrotizing hypophysitis is the rarest type of primary hypophysis, accounting for less than 1% of all primary hypophysitis [3]. Histopathologically, necrotizing hypophysitis is characterized by diffuse non-hemorrhagic necrosis with surrounding lymphocytes, plasma cells, and eosinophils [3]. Patients present with acute onset headaches with anterior or panhypopituitarism, and hormone replacement is generally required. On MRI, necrotizing hypophysitis usually appears as a diffusely enlarged pituitary gland and infundibulum, with a typical loss of central contrast enhancement (Fig. 8) [20, 21].

### Hypophysitis as immune-related adverse events (irAE)

**Fig. 9:**
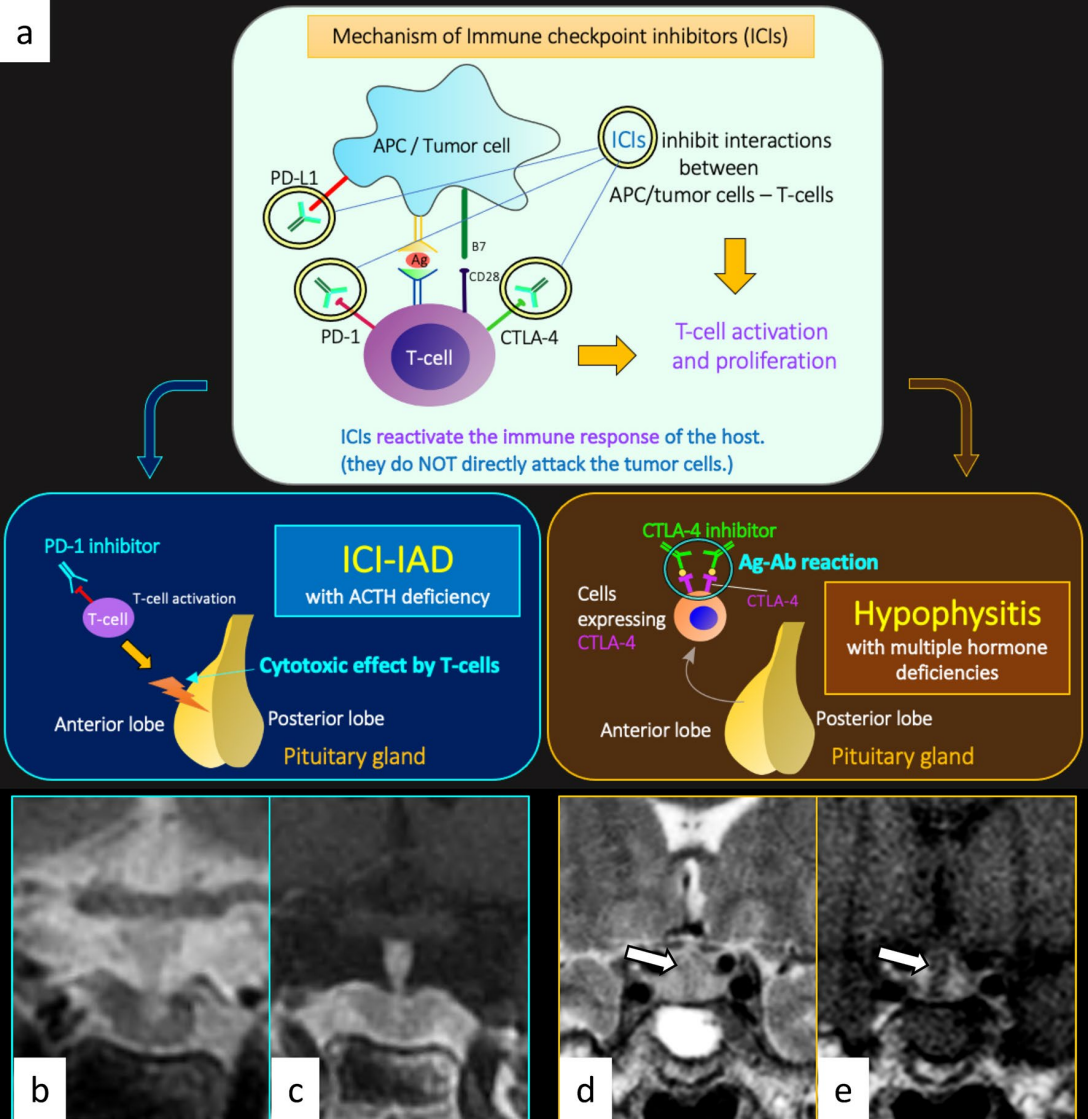
Schematic illustrations of mechanisms of immune checkpoint inhibitors (ICIs), ICI-related isolated adrenocorticotropic hormone deficiency (ICI-IAD), and ICI-related hypophysitis (**a**). ICIs inhibit interactions between antigen-presenting cells (APC)/tumor cells and T cells. ICIs include PD-1 inhibitors, PD-L1 inhibitors, and CTLA-4 inhibitors. ICIs reactivate the immune response of the host, resulting in T-cell activation and proliferation. ICI-IAD is mainly caused by the cytotoxic effect of activated T cells due to PD-1 inhibitors, whereas ICI-related hypophysitis is typically caused by antigen (Ag)-antibody (Ab) reaction due to CTLA-4 inhibitors. **b**, **c** A 68-year-old man with ICI-IAD. Both T2-weighted coronal image **b** and post-contrast T1-weighted coronal image **c** show a normal-appearing pituitary gland. **d**, **e** A 62-year-old woman with ICI-related hypophysitis. A heterogeneously enhanced, enlarged pituitary gland with longitudinal T2-weighted hypointensity areas with poor enhancement (**d**, **e**, arrows) is observed. The different images of the same patient were evaluated in a previous study [24]

Immune checkpoint inhibitors (ICIs) activate host immunity against tumors by inhibiting immune checkpoints that suppress T-cell activity. The activated T cells by ICIs, however, occasionally infiltrate into various organs and cause excessive immune responses, irAEs. There have been two distinct forms of irAEs involving the pituitary gland (Fig. 9) [22]: ICI-related isolated adrenocorticotropic hormone deficiency (ICI-IAD) and ICI-related hypophysitis (ICI-H). ICI-IAD is mainly caused by the cytotoxic effect of activated T cells due to PD-1 inhibitors and is characterized by isolated ACTH deficiency [22]. Serum anti-pituitary antibodies have been shown to be positive before the initiation of ICI treatment in the majority of cases (11/17, 65%), and the positive rate increased after symptom onset (15/17, 88%) [22]. MRI is normal in most cases of ICI-IAD [23]. On the other hand, ICI-H is typically caused by anti-CTLA4 treatment with or without combined anti-PD1 treatment, and can cause variable endocrine dysfunctions including ACTH deficiency [22]. The serum anti-pituitary antibodies were negative before ICI treatment (0/5, 0%), whereas they were frequently positive after the onset of ICI-H (4/5, 80%) [22]. On MRI, ICI-H is characterized by enlargement of the pituitary gland and stalk. Heterogeneous hypointensity on T2-weighted imaging with nodular or geographic weak and gradual enhancement on dynamic post-contrast T1-weighted imaging may be characteristic of ICI-H [24]. Patients of pituitary irAE are recommended to be managed with discontinuation of ICI until the conditions stabilize with appropriate hormone replacement and corticosteroid administration [25]. While pituitary irAE can be clinically severe, there have been reports of better survival prognosis in patients with pituitary irAE than in those without, if properly managed [26].

### Anti-pituitary-specific transcription factor 1 (PIT-1) antibody-related hypophysitis

PIT-1 is a transcription factor in the pituitary gland that plays a crucial role in the regulation of somatotroph, lactotroph, and thyrotroph differentiation during organogenesis [27]. Anti-PIT-1 antibody-related hypophysitis is an autoimmune hypophysitis associated with tumors such as thymoma, resulting in acquired selective deficiencies of growth hormone, prolactin, and TSH [28]. Pituitary MRI is usually normal, but mild pituitary atrophy was present in a few cases [28, 29].

### Differential diagnosis of hypophysitis

Imaging mimics of hypophysitis include various types of conditions including physiological change, remnant, neoplasm, and non-neoplastic lesions (Table 1).

**Table 1:** Differential diagnoses of hypophysitis

Physiological/remnant	Neoplastic	Non-neoplastic
Pituitary hyperplasia	PitNet	Sarcoidosis
Rathke's cleft cyst	Craniopharyngioma	ANCA-related vasculitis
	Pituitary metastasis	Sheehan's syndrome
	Germ cell tumor	Spontaneous intracranial hypotension
	Meningioma	
	Lymphoma	
	Pituicytoma, granular cell tumor, and spindle cell oncocytoma	
	Histiocytoma	

*PitNet* pituitary neuroendocrine tumor, *ANCA* antineutrophil cytoplasmic antibody

### Pituitary hyperplasia

**Fig. 10:**
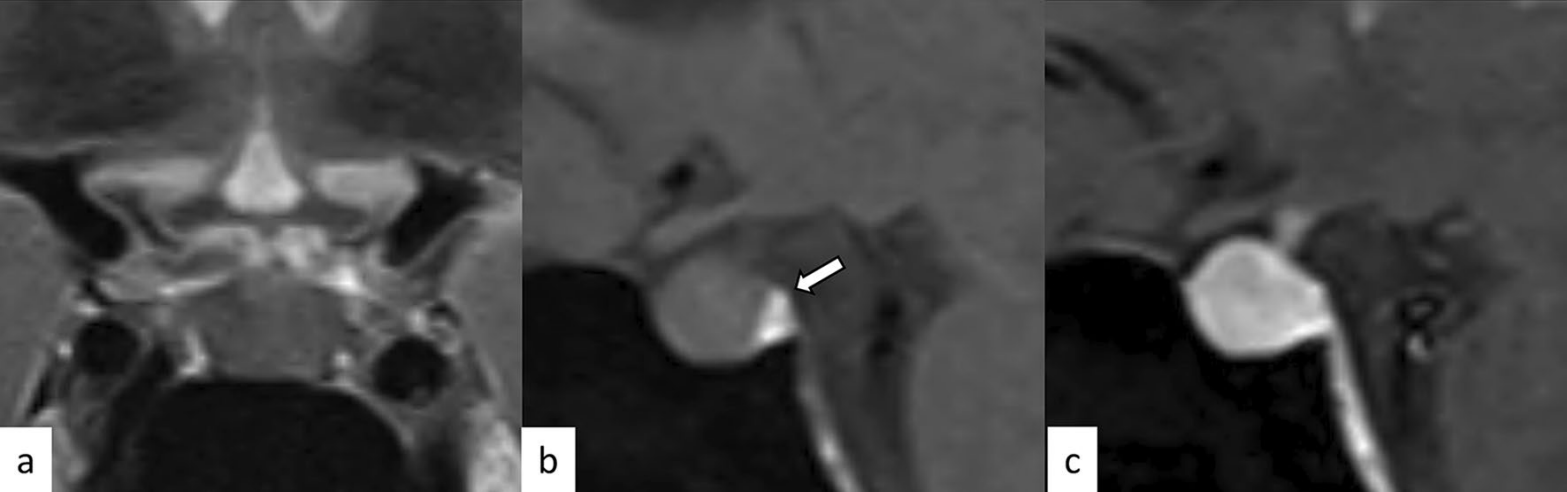
A 64-year-old woman status post-oophorectomy with pituitary hyperplasia. The pituitary gland is symmetrically enlarged with normal signal intensity on T2- and T1-weighted images **a**, **b** and homogeneous contrast enhancement (**c**). The posterior pituitary T1-weighted bright spot is preserved (**b**, arrow)

Normal pituitary size is influenced by age and sex. The upper limit of normal pituitary height is as follows: 6 mm for infants and children; 8 mm for males and postmenopausal women; 10 mm for young females; and 12 mm for pregnant or lactating women [30]. Pituitary hyperplasia is an absolute increase in the number caused by physiological, pathological, or iatrogenic conditions. Physiological pituitary hyperplasia occurs in young females and pregnant/lactating women as mentioned above. Pathological pituitary hyperplasia is associated with endocrine disorders not attributed to the pituitary gland, for example, hypothyroidism (causing thyrotroph hyperplasia), hypogonadism (causing gonadotroph hyperplasia), and excess growth hormone-releasing hormone or corticotrophin-releasing hormone (causing somatotroph or corticotroph hyperplasia, respectively) [31]. Iatrogenic hyperplasia is caused by several conditions including estrogen and antipsychotic use, both causing lactotroph hyperplasia, and gonadotrophin-releasing hormone analogs, causing gonadotroph hyperplasia [31]. On MRI, diffuse and symmetrical pituitary enlargement with homogeneous contrast enhancement is observed (Fig. 10).

### Rathke’s cleft cyst

**Fig. 11:**
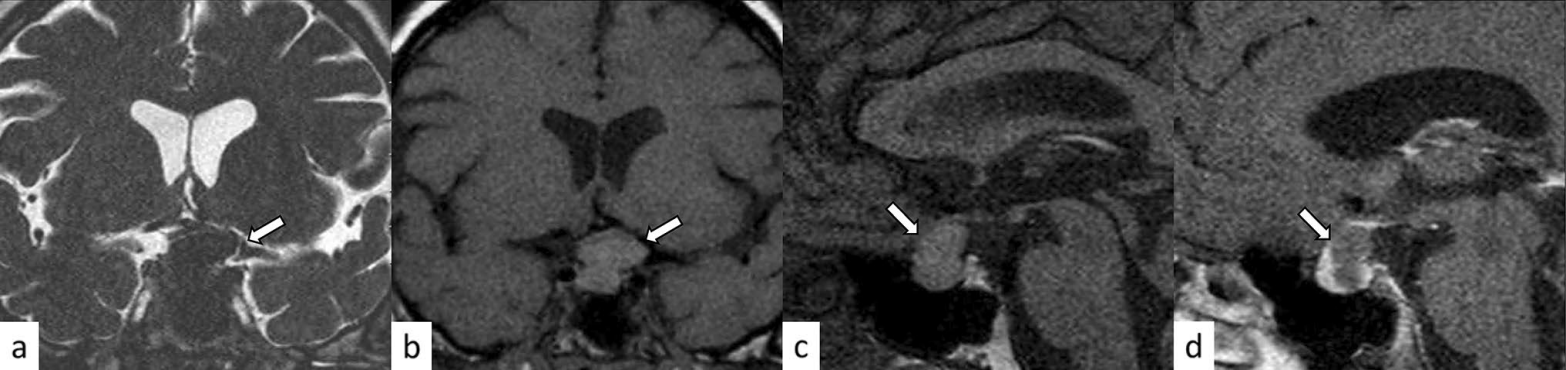
A 24-year-old woman with Rathke’s cleft cyst. There is a sellar/suprasellar mass (**a**–**d**, arrows) between the anterior and posterior lobes of the pituitary gland with hypointensity on T2-weighted coronal image (**a**) and hyperintensity on T1-weighted images **b**, **c** without contrast enhancement (**d**)

Rathke’s cleft cysts are benign sellar or suprasellar lesions originating from the remnants of Rathke’s pouch, which usually disappear during the embryonic period. The incidence of Rathke’s cleft cysts in reported 12–33% in autopsy series [32]. They are often classified as incidentalomas, found on routine MR evaluations of the brain. Cysts are typically small and are intraglandular, whereas larger, suprasellar cysts are frequently symptomatic. The incidence of symptomatic cases varies depending on the studies [32–34]. Symptoms usually result from compression of the optic pathway, hypothalamus, or pituitary gland, and may require surgical decompression. Rathke’s cleft cyst is not only a differential diagnosis of hypophysitis, but can also cause secondary hypophysitis [35–37]. Rathke’s cleft cysts occasionally present with hemorrhage, potentially mimicking hypophysitis and pituitary apoplexy [38]. On MRI, Rathke’s cleft cysts typically arise between the anterior and intermediate lobes of the pituitary gland. The cystic components show hyper- or hypointensity on T1-weighted imaging and hyper- or iso-to hypointensity on T2-weighted imaging without contrast enhancement of the wall (Fig. 11) [39]. The intracystic nodules (“waxy nodule”) can be found in the majority of cases, which appear as hyperintensity on T1-weighted imaging and hypointensity on T2-weighted imaging [34]. Shaggy wall enhancement with a consistent clinical course may indicate secondary hypophysitis [36].

## Neoplastic lesion

### PitNET/pituitary adenoma

**Fig. 12:**
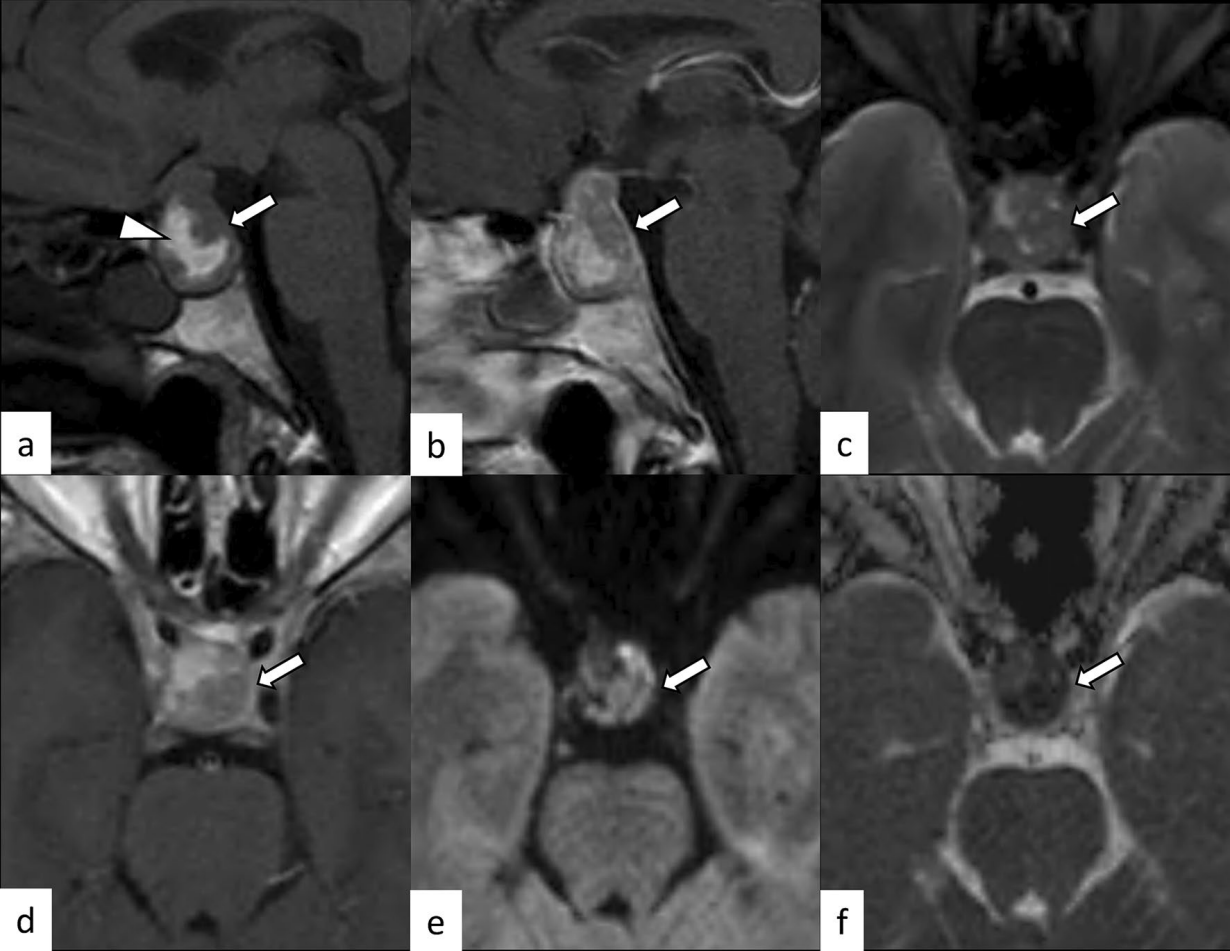
A 33-year-old woman with PitNET causing pituitary apoplexy. MRI shows a snowball-shaped mass extending from within the sella turcica to the suprasellar region (**a**–**f**, arrows). The mass has non-enhancing T1-weighted hyperintense components (**a**, arrowhead), suggestive of hemorrhagic changes, and enhancing peripheral components with T2-weighted isointensity **c** and restricted diffusion (**e**, **f**)

PitNETs are clonal neoplastic proliferations of anterior pituitary hormone-producing cells [40, 41]. PitNET is found in approximately 1 case per 1000 general population [42], and approximately 5% of cases are diagnosed before 20 years of age [40]. The term PitNET has been added to the commonly used term “pituitary adenoma” in both the WHO 2021 classification of CNS tumors and the WHO classification of Endocrine and Neuroendocrine Tumors to reflect the neuroendocrine basis of these lesions [40, 43]. Further classifications based on size (microadenoma and macroadenoma) and function (secretory and non-secretory) are commonly employed. Large PitNETs may cause compressive symptoms and various types of hypopituitarism, mimicking hypophysitis. Gutenberg et al. [44] reported that younger age ≤ 30 years, pregnancy, medium or high gadolinium contrast enhancement, loss of posterior pituitary T1-weighted bright spot (indicating loss of accumulation of neurosecretory granules including vasopressin [45]), and enlarged pituitary stalk were more suggestive of hypophysitis than PitNET. Although hypophysitis secondary to PitNET is unusual [46], PitNET occasionally causes acute hemorrhagic necrosis, resulting in pituitary apoplexy, mimicking hypophysitis (Fig. 12).

### Adamantinomatous craniopharyngioma and papillary craniopharyngioma

**Fig. 13:**
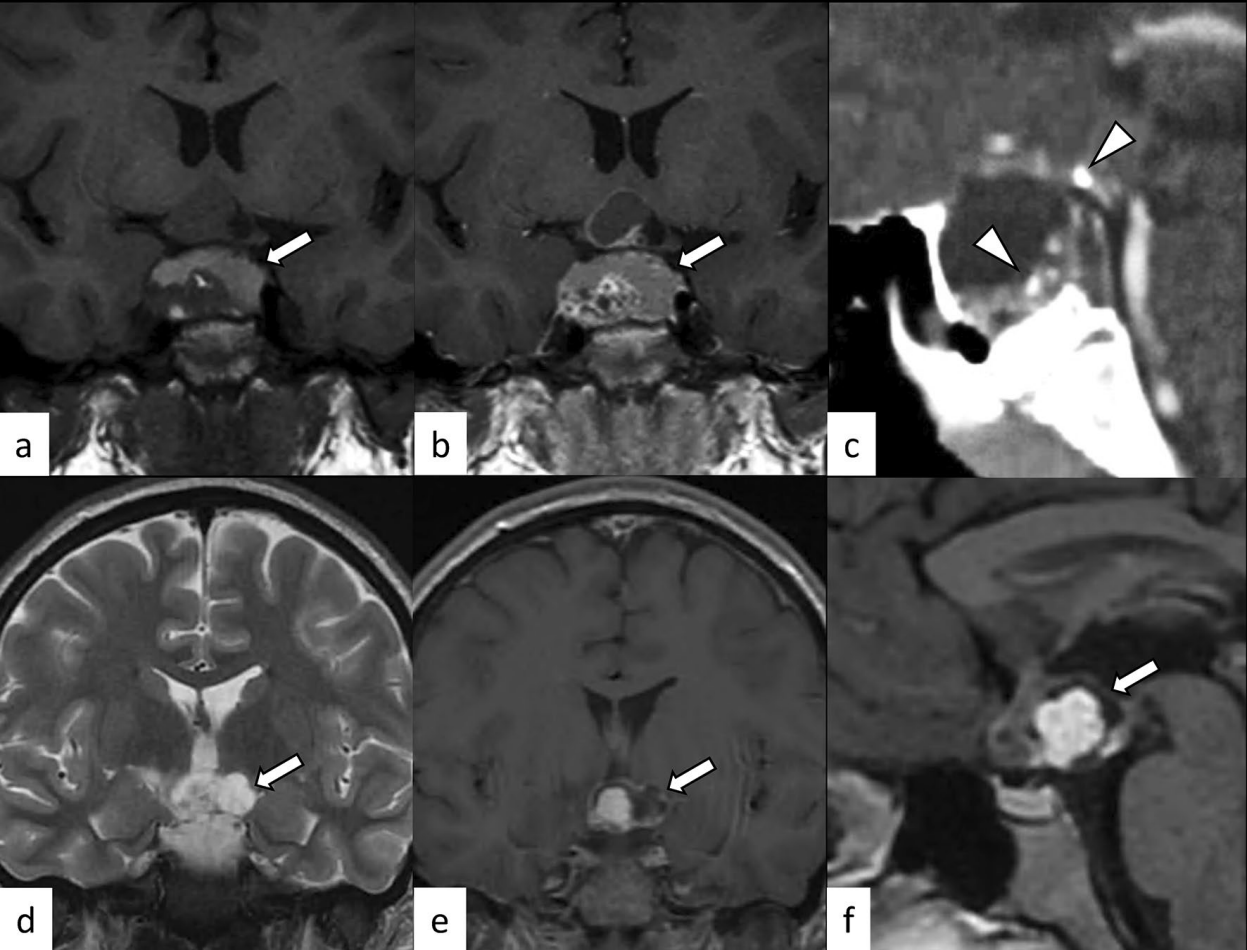
**a**–**c**, arrows A 14-year-old boy with adamantinomatous craniopharyngioma. MRI shows a mass with heterogeneous cystic signal intensity with contrast-enhanced components (**a**, T1-weighted coronal image; **b** post-contrast T1-weighted coronal image). Calcifications are observed on contrast-enhanced CT (**c** arrowheads). (**d**–**f**, arrows) A 21-year-old man with papillary craniopharyngioma. There is a suprasellar mass with heterogeneous high-to isointense solid components on T2-weighted coronal image **d** with strong contrast enhancement **e**, **f** and peripheral multicystic components

Craniopharyngiomas are CNS WHO grade 1 tumors originating from the embryonic remnants of Rathke’s pouch epithelium. There are two distinct craniopharyngiomas: adamantinomatous and papillary craniopharyngioma, both of which are regarded as CNS WHO grade 1 in 2021 WHO classification [40]. Although these craniopharyngiomas have been regarded as subtypes in the previous WHO classifications, they are now recognized as distinct tumor types based on the distinct histologies and driver mutations [40, 47, 48]. Adamantinomatous craniopharyngioma is a mixed solid and cystic squamous epithelial tumor with stellate reticulum and wet keratin, characterized by activating *CTNNB1* mutations [40]. They mainly affect children with an incidence peak in 5–15 years, accounting for nearly all craniopharyngiomas in children, while another incidence peak in 45–60 years is known [40]. Papillary craniopharyngioma is typically a solid, non-keratinizing squamous epithelial tumor, characterized by *BRAF p.V600E* mutations, and most often occurs in adults with exceptional pediatric cases [40]. Clinically, craniopharyngiomas mimic hypophysitis with frequent intracranial pressure (e.g., headaches and nausea), visual impairment, and endocrine dysfunction [49]. On neuroimaging, the adamantinomatous type typically shows a cauliflower-like shape, a solid and cystic appearance with cholesterol-rich oily fluid, vivid enhancement, and frequent calcification (Fig. 13) [49]. On the other hand, the papillary type usually develops in the suprasellar region and is vividly enhanced as mostly solid mass without calcifications [40, 49]. These imaging features may help distinguish craniopharyngiomas from hypophysitis. It should be noted, however, that craniopharyngiomas, seemingly almost always the adamantinomatous type, may cause secondary hypophysitis [50, 51].

### Pituitary metastasis

**Fig. 14:**
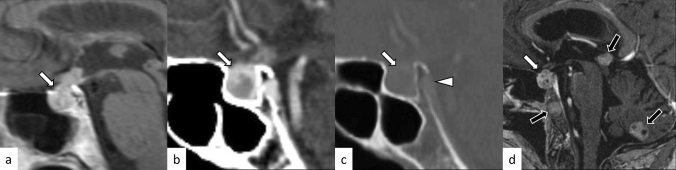
(**a**–**c**, arrows) A 76-year-old woman with pituitary metastasis of breast cancer. Post-contrast T1-weighted sagittal image shows heterogeneously enhanced mass in the pituitary gland and stalk (**a**). Contrast-enhanced CT shows the mass extending posteriorly through the clivus with evidence of bone destruction (**b**, **c**, arrowhead). (**d**, arrow) A 60-year-old woman with pituitary metastasis of small cell lung cancer. There are multiple other metastatic lesions involving the pineal region, cerebellum, and clivus (**d**, black arrows)

Pituitary metastasis from other malignancies accounts for 1.0–3.6% of surgically treated pituitary lesions and as high as 28% of autopsy cases [52]. The prognosis is unfavorable with a median survival time of approximately 6 months [52]. The most common primary sites include breast, lung, kidney, and prostate [52]. Diabetes insipidus/arginine vasopressin deficiency is a frequent symptom owing to the posterior lobe receiving direct systemic blood flow. Approximately, half of the cases arise in the posterior lobe, one-third involve both the anterior and posterior lobes, and only 15% involve the anterior lobe alone [53]. On neuroimaging, rapid enlargement, multiple lesions, and adjacent bone destruction are indicative of pituitary metastasis (Fig. 14). Bone destruction with a normal sellar size indicates the rapid growth of the tumor, in contrast to slow-growing PitNET. Contrast enhancement tends to be avid, and flow voids can be observed within the lesion [52]. However, it is often challenging to diagnose a pituitary metastasis without other metastatic lesions or bone destruction on the first surveillance MRI of the brain [54].

### Germ cell tumor

**Fig. 15:**
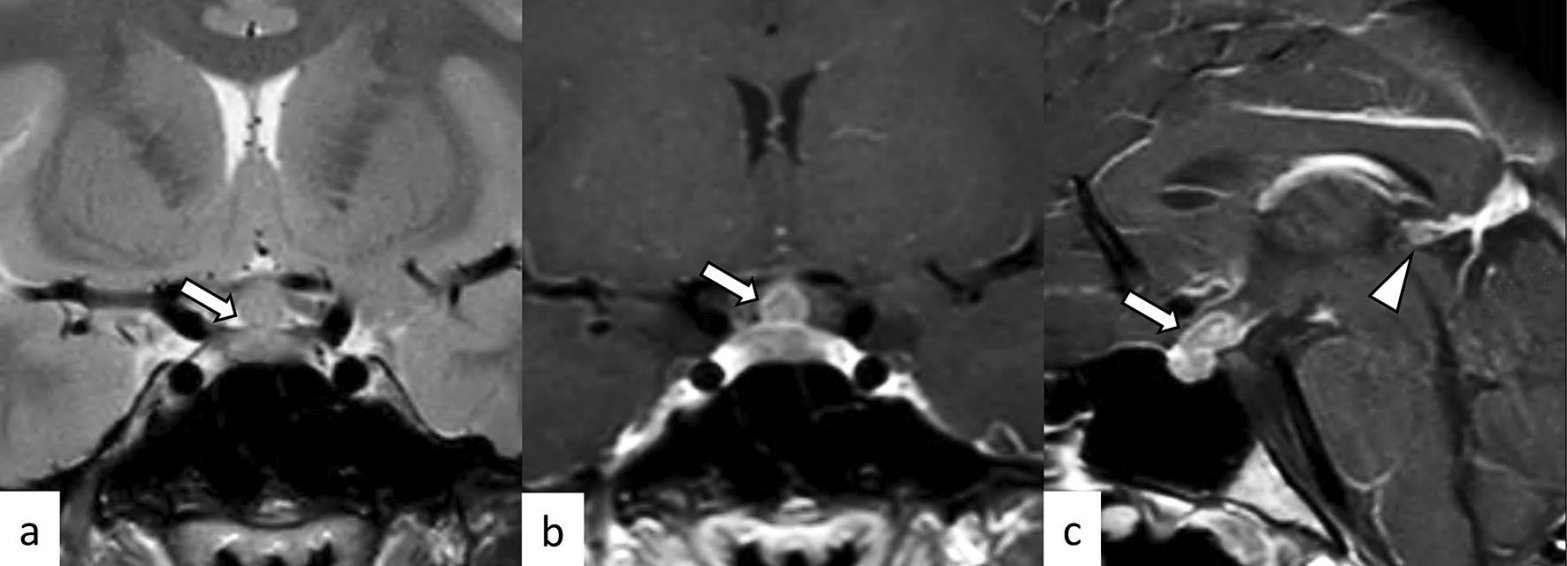
A 17-year-old woman with germinoma involving the pituitary gland and pineal gland. MRI shows enlarged pituitary gland and stalk with T2-weighted hyperintensity (**a**, arrow) and heterogeneous enhancement (**b**, **c**, arrows). A pineal gland lesion is also demonstrated (c, arrowhead)

Germ cell tumors mainly occur in pediatric and young adult patients, and sellar/suprasellar involvement accounts for 32–42% of intracranial germ cell tumors [55, 56]. The most common presenting symptom is diabetes insipidus/arginine vasopressin deficiency, accounting for more than 90% of sellar/suprasellar germ cell tumors and 75% of bifocal (i.e., sellar/suprasellar and pineal regions) germ cell tumors [55]. Germ cell tumors, especially neurohypophyseal germinoma, could be infiltrated by abundant lymphocytes or plasma cells, mimicking hypophysitis [57, 58]. Elevated serum or CSF biomarkers including alpha-fetoprotein, beta-human chorionic gonadotropin, and placental alkaline phosphatase are suggestive of germ cell tumors, but the biomarker that tends to be elevated differs depending on the included tumor subtypes [59]—elevated alpha-fetoprotein: immature teratoma, embryonal carcinoma, and yolk sac tumor; elevated beta-human chorionic gonadotropin: choriocarcinoma and some germinoma and embryonal carcinoma cases; elevated placental alkaline phosphatase: germinoma. Neuroimaging findings vary depending on the subtypes and percentage of subtypes included. The tumors show laminar, nodular, or lobular morphology, presenting as a suprasellar mass with or without extending into the sella turcica and the floor of the third ventricle (Fig. 15) [55]. Germinoma typically shows hyperdensity on CT and restricted diffusion on MRI [48, 60]. Contrast enhancement tends to be heterogeneous in mixed germ cell tumors.

### Other neoplastic lesions

**Fig. 16:**
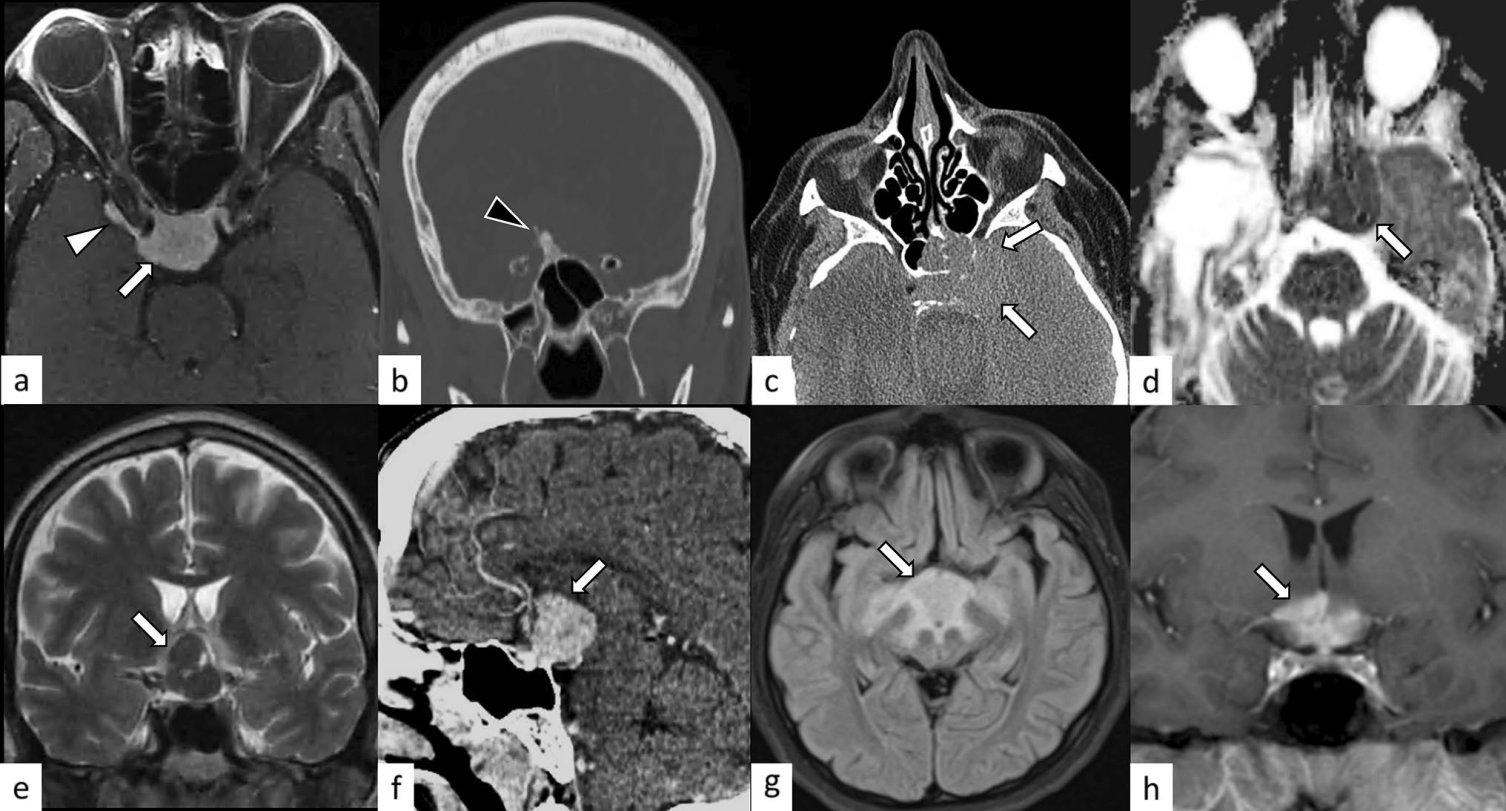
**a**, **b** A 49-year-old man with meningioma. Post-contrast fat-suppressed T1-weighted image shows homogeneously enhanced suprasellar mass (**a**, arrow). Dural-tail sign (**a**, white arrowhead) and hyperostosis (**b**, black arrowhead) are observed. **c**, **d** A 60-year-old woman with lymphoma. Non-enhanced CT shows an infiltrative hyperdense suprasellar mass (**c**, arrowheads), which shows restricted diffusion with low apparent diffusion coefficient (**d**, arrow). **e**, **f** A 51-year-old man with pituicytoma. T2-weighted coronal image shows a suprasellar well-defined mass with homogeneous hypointensity and small cystic areas (**e**, arrow). Contrast-enhanced CT shows homogeneous enhancement (f, arrow). **g**, **h** A 14-year-old girl with Rosai–Dorfman disease. FLAIR image shows an infiltrative suprasellar mass with an ill-defined margin, involving the midbrain and bilateral thalami (**g**, arrow). Post-contrast T1-weighted image shows heterogeneous and strong enhancement (**h**, arrow)

Other neoplastic lesions located in the sellar/suprasellar may masquerade as hypophysitis, both clinically and radiologically. These include meningioma, lymphoma, pituicytoma, granular cell tumor of the sellar region, and spindle cell oncocytoma, and histiocytosis (Fig. 16). Meningioma is a well-defined, extra-axial mass, and dural-tail sign and hyperostosis of adjacent bone are characteristic when present [61, 62]. Sellar/suprasellar lymphoma tends to be misdiagnosed as PitNET, lymphocytic hypophysitis, Langerhans cell histiocytosis, and metastases before pathological diagnosis [63]. On MRI, tumors show hypo- to isointensity on T1- and T2-weighted images with low apparent diffusion coefficient, located in the sellar or suprasellar regions with or without the involvement of the cavernous sinus and the sphenoid sinus [63, 64]. Pituicytoma, granular cell tumor of the sellar region, and spindle cell oncocytoma have been considered to represent a spectrum of a single nosological entity, all showing *TTF1* expression [40]. These tumors usually arise along the posterior pituitary and infundibulum, and MRI typically shows a well-defined, T2-weighted heterogeneous and hypo- to isointense mass with vivid contrast enhancement. Sellar/suprasellar histiocytosis may be observed as a meningioma-like or an infiltrative mass with elevated inflammatory markers [65, 66].

## Non-neoplastic lesion

### Sarcoidosis

**Fig. 17:**
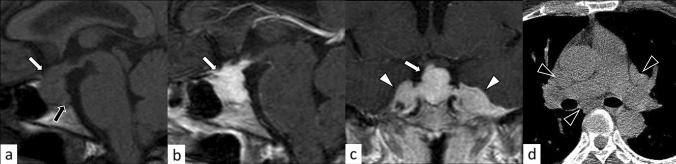
A 53-year-old woman with sarcoidosis. Enlarged pituitary gland and stalk with homogeneous and strong contrast enhancement are observed (**a**–**c**, white arrows). The posterior pituitary T1-weighted bright spot is absent (**a**, black arrow). The lesion is extending into the bilateral cavernous sinus (**c**, white arrowheads). Chest CT shows mediastinal lymphadenopathy, highly suggestive of sarcoidosis (**d**, black arrowheads)

Hypothalamo-pituitary sarcoidosis accounts for less than 1% of all intrasellar lesions [67]. Most patients present with hypopituitarism, and other clinical manifestations may be found depending on the sites involved [67]. On MRI, sellar/suprasellar/hypothalamus plaque-like or nodular lesions showing T2-weighted hyperintensity with intense contrast enhancement is observed in the active lesions, while hypointensity on T2-weighed imaging with weak contrast enhancement is observed in the chronic fibrous lesions (Fig. 17) [68]. Other lesions of neurosarcoidosis were associated in more than half of the cases: brain parenchymal, meningeal, or spinal lesions [67]. Hydrocephalus may be found in 5–12% of patients [68]. Mediastinal and hilar lymphadenopathy are frequent and may help with diagnosis. Both clinical and imaging findings can improve or disappear with corticosteroid treatment [67].

### Granulomatosis with polyangiitis (GPA)

**Fig. 18:**
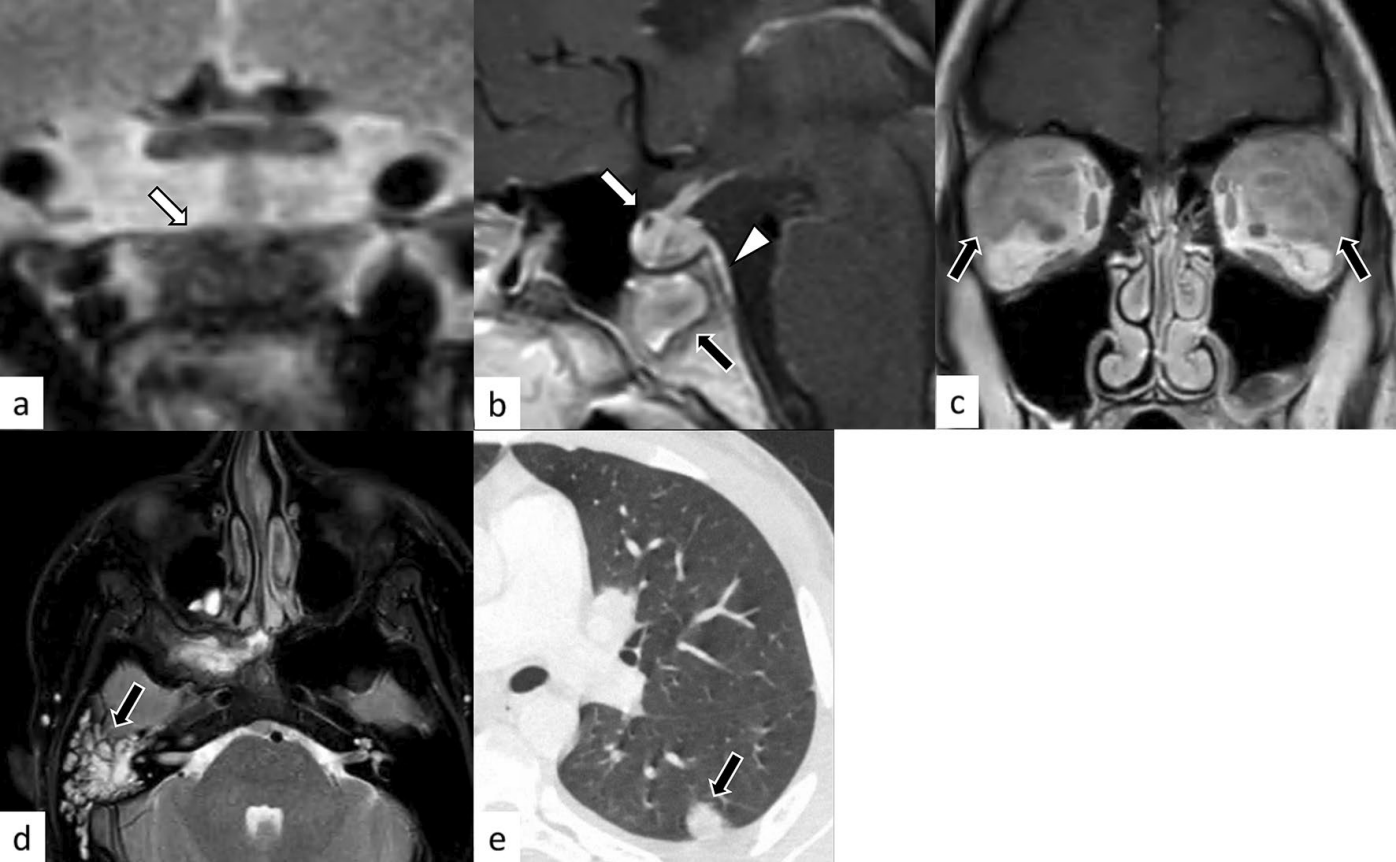
A 20-year-old man with proteinase 3-antineutrophil cytoplasmic antibody-positive GPA. The pituitary gland is enlarged and shows heterogeneously hypointensity on T2-weighted coronal image (**a**, white arrow) with heterogeneous enhancement (**b**, white arrow), extending posteriorly along the surface of the clivus (**b**, white arrowhead). The sphenoid sinus (**b**, black arrow), bilateral lacrimal glands (**c**, black arrows), right mastoiditis (**d**, black arrow), and left lung (**e**, black arrow) are also involved

The pituitary gland is involved in up to 1.3% of patients with GPA [69, 70]. The pituitary lesion may be a distant granuloma or direct spread from nasal, paranasal, or orbital disease [70]. The vasculitic components of the disease are infrequently observed, compared with granulomatous components [70]. Although pituitary involvement can be the initial presentation, it usually occurs during the course of other GPA lesions within an interval from months to years after diagnosis [69]. Like other mimickers of hypophysitis, GPA may cause compressive symptoms (e.g., headaches, nausea, visual impairment) and endocrine dysfunction [69, 70]. On MRI, the pituitary lesion typically shows hypointensity on T2-weighted imaging with heterogeneous contrast enhancement (Fig. 18). Other findings, such as nasal, paranasal, orbital, mastoid, meningeal, and pulmonary involvements may be diagnostic indicators. Both clinical and imaging findings can improve or disappear with corticosteroid and/or immunosuppressive agents, although hypopituitarism tends to be persistent, and long hormonal replacement is usually required [69].

### Sheehan’s syndrome

**Fig. 19:**
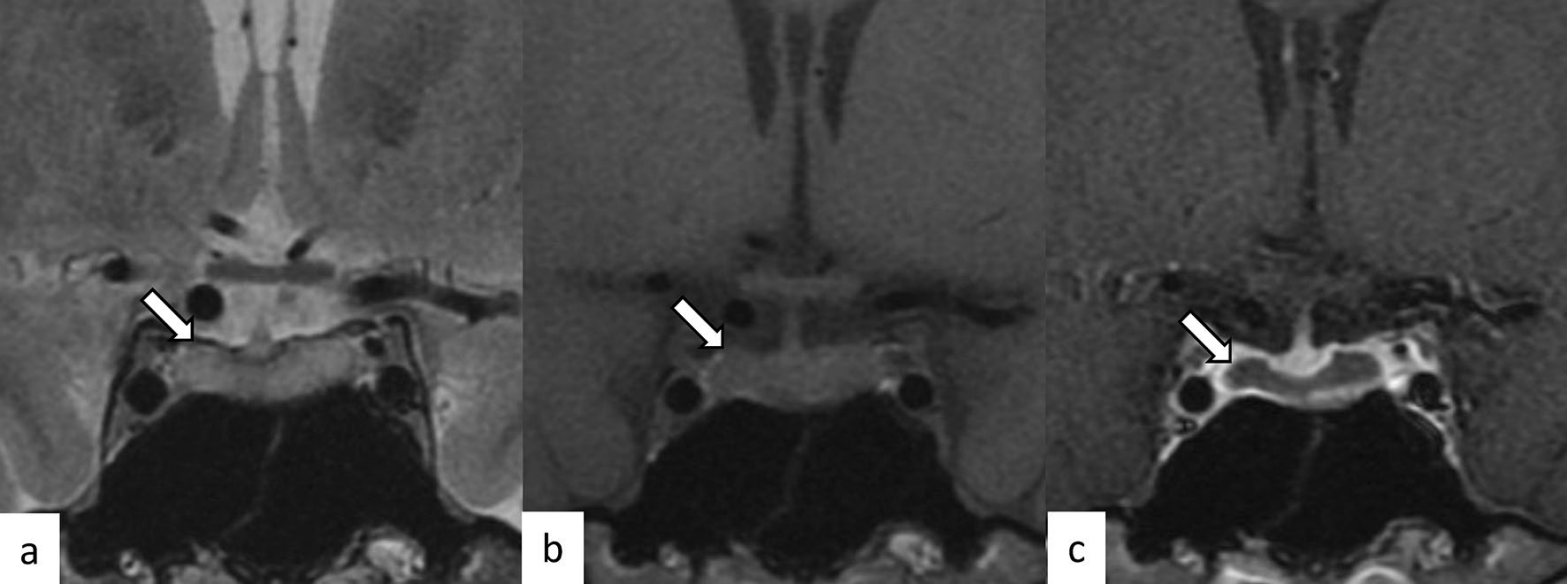
A 33-year-old woman with Sheehan’s syndrome after a cesarean section with massive blood loss. The size of the pituitary gland is within normal limits. The anterior pituitary lobe shows hyperintensity on T2-weighted coronal image (**a**, arrow), isointensity on T1-weighted coronal image (**b**, arrow) without contrast enhancement (**c**, arrow)

Sheehan’s syndrome is hypopituitarism due to postpartum pituitary necrosis after massive postpartum hemorrhage and severe hypotension. Hyperplasia of lactotrophs and other anterior pituitary gland cells during pregnancy causes the increased size of the anterior pituitary lobe, which is susceptible to ischemia [71]. Therefore, massive bleeding and associated hypotension tend to trigger pituitary necrosis. Patients with Sheehan’s syndrome show anterior or panhypopituitarism in either acute or chronic phases. A detailed perinatal medical history is necessary to differentiate Sheehan’s syndrome from differential diagnoses such as lymphocytic hypophysitis and pituitary apoplexy due to other causes. On MRI, weak or absence of contrast enhancement is observed (Fig. 19) [72]. Follow-up MRI may demonstrate empty sella [72].

### Spontaneous intracranial hypotension (SIH)

**Fig. 20:**
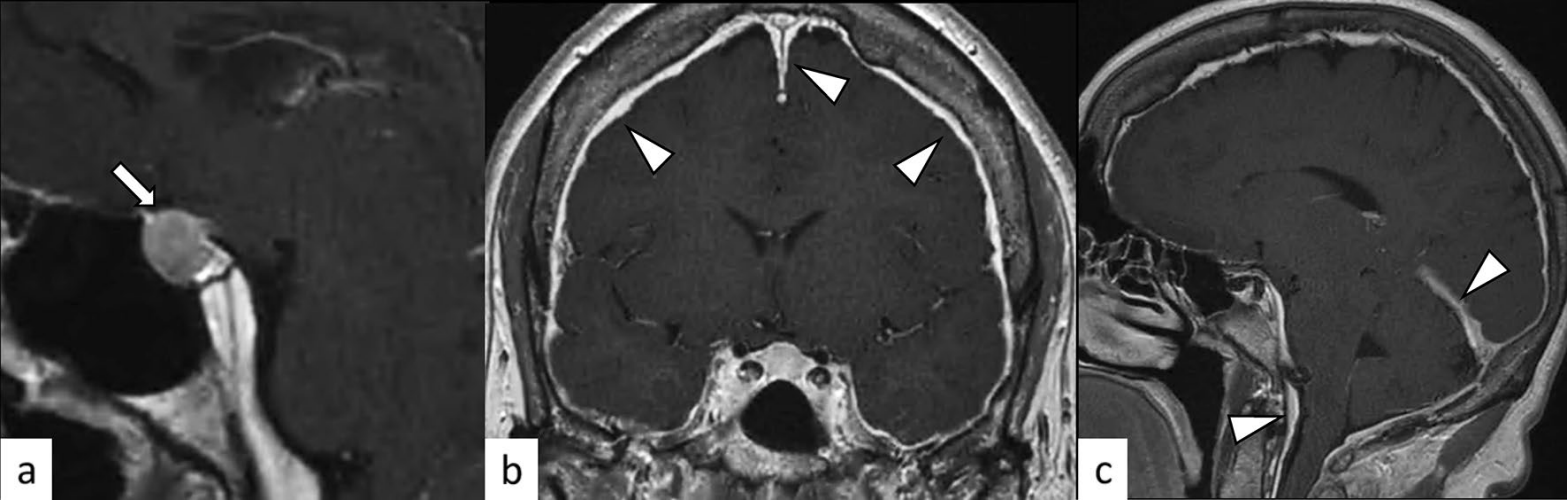
A 62-year-old woman with spontaneous intracranial hypotension. An enlarged pituitary gland is observed on post-contrast T1-weighted sagittal image (**a**, arrow). Diffuse dural thickening and linear enhancement in both supra- and infratentorial regions are observed (**b**, **c**, white arrowheads). A slight downward displacement of the structures appears near the base of the brain, including the hypothalamus, midbrain, pons, and cerebellum

SIH is caused by CSF volume depletion, which typically results from CSF leakage at the lower cervical and upper thoracic spine [73]. A female predominance with a peak incidence around the fourth decade has been known [73]. SIH is clinically characterized by orthostatic headache, nausea/vomiting, neck rigidity, and cranial nerve palsies [74]. On MRI, an enlarged pituitary gland is observed (Fig. 20) [75]. Other MRI features, including pachymeningeal thickening and enhancement, brain sagging, and bilateral subdural effusion, may help in accurate diagnosis.

## Conclusion

Herein, we have reviewed the clinical and imaging features of primary and secondary hypophysitis and their various mimickers. The recognition of both clinical and imaging findings, including those at other sites in the body, may provide diagnostic indicators and facilitate appropriate management.
